# Use of latent class models to accommodate inter-laboratory variation in assessing genetic polymorphisms associated with disease risk

**DOI:** 10.1186/1471-2156-9-51

**Published:** 2008-08-08

**Authors:** Stephen D Walter, Eduardo L Franco

**Affiliations:** 1 Department of Clinical Epidemiology and Biostatistics, McMaster University, Hamilton, Ontario, Canada; 2 Departments of Oncology and Epidemiology & Biostatistics, McGill University, Montreal, Quebec, Canada

## Abstract

**Background:**

Researchers wanting to study the association of genetic factors with disease may encounter variability in the laboratory methods used to establish genotypes or other traits. Such variability leads to uncertainty in determining the strength of a genotype as a risk factor. This problem is illustrated using data from a case-control study of cervical cancer in which some subjects were independently assessed by different laboratories for the presence of a genetic polymorphism. Inter-laboratory agreement was only moderate, which led to a very wide range of empirical odds ratios (ORs) with the disease, depending on how disagreements were treated.

This paper illustrates the use of latent class models (LCMs) and to estimate OR while taking laboratory accuracy into account. Possible LCMs are characterised in terms of the number of laboratory measurements available, and if their error rates are assumed to be differential or non-differential by disease status and/or laboratory.

**Results:**

The LCM results give maximum likelihood estimates of laboratory accuracy rates and the OR of the genetic variable and disease, and avoid the ambiguities of the empirical results. Having allowed for possible measurement error in the expure, the LCM estimates of exposure – disease associations are typically stronger than their empirical equivalents. Also the LCM estimates exploit all the available data, and hence have relatively low standard errors.

**Conclusion:**

Our approach provides a way to evaluate the association of a polymorphism with disease, while taking laboratory measurement error into account. Ambiguities in the empirical data arising from disagreements between laboratories are avoided, and the estimated polymorphism-disease association is typically enhanced.

## Background

This paper was motivated by a study in which a putative genetic risk marker for disease could not be measured with certainty. The study used a case-control design to assess the association of cervical cancer with a polymorphism in codon 72 of the p53 tumour suppressor gene. DNA specimens from study participants were processed independently and blindly to disease status by three laboratories in different countries. Preliminary analyses showed that inter-laboratory agreement on the genotype was only moderate, which led to considerable ambiguity about its odds ratio (OR) with cervical cancer [1]. The empirical estimates of OR varied widely, depending on how disagreements between laboratory results were treated.

Statistical latent class models (LCM) have been applied to a wide variety of diagnostic or disease screening data where disease status cannot be established with certainty. Typical scenarios are where a gold standard classification of disease either does not exist or is infeasible to observe [2-17]. The goal of LCM is typically to estimate measurement properties (such as test sensitivities and specificities) of the imperfect methods that are used to assess disease status. These ideas have been applied to meta-analyses as well as to individual studies [18]. In contrast, the motivating case-control study on cervical cancer involved uncertainty about the genetic risk factor, rather than about disease status.

Our illustrative example was a hospital-based case-control study of cervical cancer and the p53 codon 72 polymorphism, carried out in Brazil [1], where the cases had histologically confirmed invasive squamous cell carcinoma of the cervix. Controls were sampled from women who attended a cervical cancer screening program in the same hospital where the cases were seen. Absence of malignancy in the controls was based on cytological examination of Pap smear samples. p53 codon 72 genotyping was performed blindly by 3 independent laboratories in Montreal, Canada, Sao Paulo, Brazil, and London, UK, randomly labelled here as laboratories A, B and C.

Misclassification of disease status for the cases was unlikely because histological confirmation of squamous carcinoma was required. Although cervical abnormalities may have existed in previous Pap smears from control women, it is unlikely that any controls would have undetected cervical cancers at the time of study enrolment, because these invasive lesions would have been detected upon examination. To guard against false negatives on cytology, Pap smears from control women were read twice by independent expert cytopathologists [1].

In order to investigate inter-laboratory variation in test results, a random sample of participants was drawn by an epidemiology team in Montreal and submitted to the Sao Paulo centre, where the DNA specimens were stored. Specimens for selected women were divided into three aliquots, with two being shipped on dry ice to Montreal and London. The laboratories independently reported their classifications of the polymorphism to the epidemiologists in Montreal. Technical details of the laboratory methods varied, as previously described [1].

The study was not originally designed to assess the association between polymorphism and disease risk, because the index publication on the potential utility of this risk marker appeared several years after it was conducted [19]. However, given the availability of stored specimens for many of the subjects, the authors decided to test the hypothesis on a *post-hoc *basis. The original report of this study included 54 cases and 91 controls. Pairwise comparisons between laboratories indicated crude agreement ranging from 71% to 78%, and chance-corrected kappa statistics of 0.49 to 0.63, implying moderate to substantial inter-laboratory reliability [20]. The fact that pairs of results disagree quite frequently (about 25% of the time) underscores the problem of not having a clear-cut definition of how a given woman should be classified if disagreements arise. Table 1 shows crude and age- and race-adjusted ORs, associated with the homozygous Arg/Arg genotype, vs. a reference category of heterozygous Arg/Pro and homozygous Pro/Pro genotypes combined.

**Table 1:** Association of invasive cervical cancer with p53 arg/arg genotype^1 ^using individual laboratory results

	**OR^1 ^(95% CI)**	
	**Crude**	**Adjusted**^2^
Laboratory A	2.5 (1.1–5.6)	3.2 (1.3–7.9)
Laboratory B	2.2 (1.0–5.1)	2.4 (1.0–5.9)
Laboratory C	1.8 (0.7–4.8)	2.8 (0.9–8.4)

^1 ^Odds ratio (OR) relative to reference category combining Arg/Pro and Pro/Pro genotypes.

^2 ^OR adjusted for age and race.

Faced with the apparent unreliability of the laboratory results, the study investigators adopted alternative definitions of the reference and index categories. For the reference category, the *non-stringent *definition permitted disagreements for the Arg/Pro and the Pro/Pro genotypes, while the *stringent *definition included only genotypes with complete agreement among the laboratories. The index category was defined as: *disagreed *when it included only those subjects with an Arg/Arg genotype result from at least one laboratory but with different results from the other laboratories; *agreed *when it included only Arg/Arg subjects with complete agreement among laboratories; or *all-inclusive *when it allowed any reported Arg/Arg genotype, with or without agreement. Table 2 shows the OR estimates associated with all 6 combinations of reference and index category definitions, obtained using unconditional logistic regression [1]. The results varied widely, leading the investigators to conclude that "*When disagreement between laboratories was allowed,...OR was as low as 1.5. In contrast, OR increased to 8.0 after exclusion of discordant genotypes ...Exposure misclassification ... may affect ability to detect the association..." *[1] The lowest of these OR values (1.5) would represent a relatively weak association between the polymorphism and cervical cancer, while the largest (8.0) would represent a rather strong association, and there is considerable ambiguity about which of any of the empirical OR values is most "correct". It should be noted that all these OR estimates, including even the estimates based on excluding the discordant observations, are biased [21], possibly quite seriously. Estimates using the data from only one laboratory are also biased in the presence of measurement error.

**Table 2:** Association of invasive cervical cancer with p53 arg/arg genotype^1^, with various approaches to inter-laboratory disagreements

**Referent category definition**^**1**^	**Index category (Arg/Arg genotype) definition**^**2**^	**OR (95% CI)**	
		**Crude**	**Adjusted**^**3**^
Non-stringent	Disagreed	1.6 (0.6–4.2)	1.5 (0.5–3.9)
	All-inclusive	2.4 (1.2–5.0)	2.4 (1.1–5.3)
	Agreed	2.6 (1.0–6.9)	3.4 (1.2–9.9)
Stringent	Disagreed	2.7 (0.9–8.1)	2.8 (0.9–8.9)
	All-inclusive	4.1 (1.7–10.0)	5.0 (1.9–13.3)
	Agreed	4.5 (1.5–13.4)	8.0 (2.3–28.5)

^1 ^Non-stringent definition allows inter-laboratory disagreement for the Arg/Pro and the Pro/Pro genotypes; stringent definition includes only genotypes with complete agreement among the three laboratories (adapted from reference 1).

^2 ^Disagreed: includes only subjects with an Arg/Arg genotype determined by at least one laboratory but with different results from the other laboratories; Agreed: includes only Arg/Arg subjects with complete agreement among laboratories; All-inclusive: includes any reported Arg/Arg genotype with or without agreement among laboratories or in isolation

^3 ^OR adjusted for age and race.

The uncertainty engendered by the wide range of these empirical estimates, and the lack of a preferred estimator motivated us to develop a LCM analysis that could assess the association of the polymorphism with disease, while taking potential inaccuracy of the laboratory results into account. Such an approach should lead to a de-attenuation of the exposure-disease association, giving a more rigorous way to estimate OR. Additionally, investigators can learn about the likely quality of their data, in terms of the accuracy rates of their contributing laboratories.

In our Methods section, we determine design requirements for the application of LCMs in this situation, according to alternative assumptions about variation in test accuracy. In our Results section, we apply several models to assess the genotype-cervical cancer association, while taking test inaccuracy into account. Use of LCMs for the problem yields maximum likelihood estimates of OR, which have superior statistical properties to the biased empirical estimates mentioned above. Other issues in the application of LCMs to this type of problem are covered in our Discussion.

## Methods

We assume that a true exposure status X exists for the genotype of each study subject, but that it cannot be observed without error – hence X is a latent or unobserved variable. We are interested in the association of disease D (cervical cancer) with the true exposure status X, denoted by DX, but instead we can only observe DE, the association of disease with the observed laboratory results E.

The accuracy of a laboratory test for a risk factor can be characterised by two measures. First, sensitivity is the probability that an individual whose true exposure X is positive receives a correct positive negative result. Second, specificity is the probability that an individual whose true exposure X is negative receives a correct positive result. The complements (1-sensitivity) and (1-specificity) of these quantities are the false-negative and false-positive rates, these being the probabilities of incorrect results for true positive and true negative individuals, respectively [22]. Our proposed LCM estimates the joint probabilities of the set of results for a study participant, conditional on an assumed true state for that individual. The conditional probabilities are then summed over the marginal probability distribution of X, which is also estimated from the data. By suitable specification of alternative models (see below), one can evaluate if accuracy varies significantly between laboratories or by disease status. Additionally we can assess the association of the latent variable with disease, under various assumptions about test accuracy.

### 3.1: Required parameters and available degrees of freedom

In our analytic framework, we are primarily concerned with two types of LCMs. First, we wish to evaluate the measurement accuracy of the exposure data, i.e. the association of the observed genotype test results with respect to the true (but latent) genotype. Here we can either assume test accuracy to be differential or constant between laboratories, between cases and non-cases, or jointly differential by both laboratory and disease status. Second, we wish to estimate the association of disease with the true genotype, and here again we may or may not assume test accuracy to be differential by laboratory and/or disease. Finally, we can compare the LCM results with empirical (non-latent) models which examine the association of disease with the observed genotypes, but which do not admit the possibility of measurement error.

Table 3 shows the number of parameters involved in each of these three types of model. This is done for a general specification of the number of laboratories (R), and also for either 1, 2 or 3 laboratories in particular. In the first group of models (models 1–4), the focus is on evaluating test accuracy, and to examine if accuracy is the same or different between laboratories and/or between cases and controls. We examine the association of the set of laboratory results E and X, either conditionally or unconditionally on disease status (D) and laboratory. If the tests are highly accurate, there will be a strong EX association.

**Table 3:** Number of parameters in latent class model, by number of laboratories (R)

**Model number**	**Association of interest**	**Accuracy differential by laboratory**	**General number of labs (R)**	**R = 1**	**R = 2**	**R = 3**
1	Observed vs. true exposure status: EX	Yes	2R + 2	4	6	8
2		Yes*	4R + 2	6	10	14
3		No	4	4	4	4
4		No*	6	6	6	6
5	Disease vs. true exposure status: DX	Yes	2R + 3	5	7	9
6		No	5	5	5	5
7	Disease with observed exposure status: DE	Yes	2R + 1	3	5	7
8		No	3	3	3	3

* error rates are (additionally) differential by disease status

D- disease; X-true genotype status; E-genotype status measured by laboratory test.

In model 1, we allow the values of test sensitivity and specificity to be different for each laboratory, but accuracy is otherwise assumed to be the same for both cases and controls. Hence if there are R laboratories, there are 2R parameters representing test accuracy. We require two additional parameters, first to fit the marginal distributions of X (the latent exposure variable) and second for D (to constrain the case and control frequencies to agree with their observed values), making 2R + 2 parameters in total. In model 2, accuracy is now additionally permitted to be differential by disease status, which increases the number of model parameters by 2 for each laboratory, giving 4R + 2 parameters in total. In models 3 and 4, accuracy is assumed to be constant (non-differential) across laboratories, and so the number of parameters is independent of the number of laboratories. For model 3, where accuracy is non-differential by disease status, there are two accuracy parameters (sensitivity and specificity, constant across laboratories), and one each for the marginal distributions of X and D as before. For model 4, the two accuracy parameters are potentially different in the case and control groups.

In the second group of models (5 and 6), we evaluate the relationship between disease and (true) exposure X, or the DX association. In the more general case (model 5), where test accuracy varies by laboratory (but is the same for cases and controls), the parameters are the same as in model 2, except that we now include a term for the conditional probability of D given X, or D|X.

In the third group (models 7 and 8), we examine the empirical association between D and E, which involves 2R parameters in the more general situation when accuracy is allowed to vary between laboratories. Additionally, we again include a D term to constrain the fitted and observed numbers of cases and controls to agree, making 2R + 1 parameters in total. If accuracy is assumed non-differential between laboratories, there are only 3 parameters – the proportion of study subjects who are cases, and the proportions of cases and controls that are exposed. Empirical models ignore the possibility of measurement error. The empirical approach is often used in practice, but the estimated DE association will in general be biased, unless the exposure assessment is error-free. If the tests are indeed perfect (an unlikely situation in practice), the empirical models suffice and the need for modelling the measurement error process is obviated.

To estimate the parameters of the various LCMs, we need to verify that there are sufficient degrees of freedom (*df*) available from the observational design. For all the models in Table 3, the cross-classification of the R laboratory results by disease status involves 2^R+1 ^data cells, implying that there are 2^R+1 ^- 1 *df *available for parameter estimation after conditioning on the total sample size. For R = 1, 2 and 3 specifically, the available *df *are 3, 7 and 15 respectively. Therefore, among the models assessing the EX association, model 2 (which allows for the most general pattern of test accuracy) requires that there be at least 3 laboratory tests. However, the other models in this group, which assume non-differential test accuracy by disease status and/or by laboratory, can be fitted if R ≥ 2.

Models 5 and 6 examining the DX association can be fitted if there are at least 2 laboratories. Finally, the empirical evaluation of the {ED association (models 7 and 8) is possible in one or more laboratories.

Note that having sufficient *df *for parameter estimation does not avoid the issue of parameter identifiability. Because, by definition, the true latent state X is unobservable, there are usually two sets of parameter estimates with the same likelihood and model fit, these being essentially "mirror images" of one another [16,23]. Thus, for instance the laboratory sensitivity in one solution can be exchanged with a corresponding value of (1-specificity) specificity in the other. In practice, choosing the "right" solution is typically straightforward, because it will have inherently far greater plausibility in terms of agreeing with external information on the parameter values. For example, an estimated sensitivity of (say) 90% would almost certainly be more plausible than a 90% false-positive rate.

Table 4 summarises the associations that are estimated in each of the models described in Table 3, for the specific case of R = 3 laboratories (as we have in our example). For instance, in model 1 the focus is on the test accuracy, through the associations of test results from laboratories A, B and C with the true genotype status X; these associations are represented by the probabilities A|X, B|X, C|X of a positive test result from each laboratory, conditional on the true value of X. We must additionally estimate the prevalence of the latent exposure variable X.

**Table 4:** Terms involved in latent class models, for R = 3 laboratories

**Model**	**Terms of interest**	**Other terms fitted**
1	Test accuracy (A|X, B|X, C|X)	True exposure prevalence (X)
2	Test accuracy, differential by disease (A|DX, B|DX, C|DX)	True exposure prevalence (X)
3	Test accuracy, constant across labs (A|X = B|X = C|X)	True exposure prevalence (X)
4	Test accuracy, constant across labs, differential by disease (A|DX = B|DX = C|DX)	True exposure prevalence (X)
5	True exposure by disease (X|D)	Test accuracy (A|X, B|X, C|X)
6	True exposure by disease (X|D)	Test accuracy, constant across labs (A|X = B|X = C|X)
7	Empirical exposure by disease (A|D, B|D, C|D)	
8	Empirical exposure by disease constant across labs (A|D = B|D = C|D)	

= : indicates terms constrained to be equal

Model 2 examines test accuracy in more detail, specific to both laboratory and disease status, by fitting the conditional probabilities A|DX, B|DX, C|DX. Models 3 and 4 impose equality constraints on the terms, to force the test accuracy estimates to be the same across laboratories.

In models 5 and 6, the focus is on the fitted term X|D that defines the association of the genotype with disease, while the models also allow for test accuracy. Finally, models 7 and 8 examine the empirical test positivity rates, conditional on disease state, through terms such as A|D; no allowance is made for the possibility of test errors.

The LCM models are actually fitted by calculating expected frequencies in the cells of the contingency table formed by a cross-tabulation of the observed variables. These expectations can be represented in a standard log-linear form. [24] For instance, for model 5, the log-linear formulation of the expected frequency for the data frequency m_abcdx_, corresponding to levels a, b, c, d, and x of the observed laboratory test variables (A, B, C), the disease status D and the latent variable X respectively, is given by

$$\ell n(m_{abcdx})=u+u_{a}^{A}+u_{b}^{B}+u_{c}^{C}+u_{d}^{D}+u_{x}^{X}+u_{ax}^{AX}+u_{bx}^{BX}+u_{cx}^{CX}+u_{dx}^{DX}$$

where *u *represents the overall mean frequency across all cells, a main effect term such as $u_{a}^{A}$ represents a marginal constraint on the frequencies at each level of A, and the interaction terms such as $u_{dx}^{DX}$ indicates that the associations such as DX are to be estimated.

We used the freeware program *lem *[25], which provides a flexible framework for latent class analysis. Latent class software, such as *lem*, more easily accommodates the type of data and modelling required for this type of analysis. Programs for the general analysis of log-linear models can also be adopted, if the user is able to specify the requisite latent class models appropriately in a corresponding log-linear format.

Comparisons between the fits of appropriate pairs of models permits evaluation of the various assumptions, such as those of differential test accuracy between laboratories and disease groups. Statistical significance of the differences in fit between alternative models can be assessed using likelihood ratio statistics.

The *lem *program allows conditioning on the observed pattern of available data, so that data from women with results available from only one or two laboratories can be used. We assume that data missingness is unrelated to the model parameters of interest, because the chance of an uninformative test result depends primarily on the degree of depletion of the DNA specimen, and not on p53 status. Model fitting is based on the EM algorithm, and iterative proportional fitting, with parameter starting values defined via a random number seed. This method of fitting yields maximum likelihood estimates of the model parameters, which are therefore unbiased in large samples, and have the smallest possible variance. These statistical properties imply strong advantages of the LCM parameter estimates, compared to the *ad hoc *estimates described earlier.

## Results

Our analysis is based on a larger sample of participants obtained subsequent to the original report [1], with 142 cases and 162 controls identified using the same methods as previously. Table 5 shows the numbers of participants with polymorphism classifications available from the various combinations of laboratories. Laboratory B did more tests because they were able to salvage additional DNA samples from the frozen cervical specimens. Laboratories varied in their diligence in obtaining informative test results, and their potential to do so also varied by the amount of fractionated sample material available to them.

**Table 5:** Number of cases and controls with p53 classifications available, by laboratory

		**Lab C**					
		**Cases**			**Controls**		
**Lab A**	**Lab B**	**Arg/Arg**	**Other**	**NA**	**Arg/Arg**	**Other**	**NA**
Arg/Arg	Arg/Arg	5	2	4	7	5	1
	Other	0	4	0	1	2	0
	NA	1	0	3	3	1	1
Other	Arg/Arg	1	0	1	0	4	0
	Other	1	12	0	1	33	2
	NA	0	1	3	1	7	4
NA	Arg/Arg	2	1	26	0	0	10
	Other	0	0	65	0	1	44
	NA	0	1	9	1	1	32

### Assessment of laboratory accuracy

Table 6 shows results from the first group of models in Table 3, examining the accuracy of the laboratory classifications of the polymorphism. Model 1 estimates the prevalence of the latent genotype X, and the probability of each laboratory result (A, B, or C) conditional on X, while conditioning on the observed number of cases and controls through inclusion of the variable D. Model 2 is similar, but it conditions the probability of laboratory results to depend on D as well as X. A likelihood ratio test between models 1 and 2 gives χ^2 ^= 9.2 on 6 *df *(p = 0.16), indicating no strong evidence of differential test accuracy between cancer cases and controls, while still allowing differential accuracy by laboratory. This is reassuring, given that DNA samples from cases tend to be more plentiful than from controls. (Case biopsy samples contain more cells than cervical cell swabs from controls). Specimens with a greater quantity of DNA permit replication of results whenever the interpretation of the first assay was uninformative.

**Table 6:** Results for latent class models focussing on laboratory error rates

**Model**	**Log- likelihood**	**Disease Groups**	**Lab**	**Sensitivity (SE)**	**Specificity (SE)**
1	-441.64	All	A	0.94 (0.07)	0.90 (0.04)
			B	0.94 (0.08)	0.94 (0.03)
			C	0.70 (0.10)	0.95 (0.05)
2	-437.06	Cases	A	0.89 (0.10)	0.76 (0.10)
			B	1.00 (0.00)	0.97 (0.07)
			C	0.77 (0.15)	0.94 (0.06)
		Controls	A	1.00 (0.00)	1.00 (0.00)
			B	0.73 (0.11)	0.93 (0.03)
			C	0.59 (0.11)	0.95 (0.03)
3	-445.94	All	A	0.83 (0.06)	0.93 (0.02)
			B	0.83 (0.06)	0.93 (0.02)
			C	0.83 (0.06)	0.93 (0.02)
4	-443.56	Cases	A	0.90 (0.08)	0.88 (0.05)
			B	0.90 (0.08)	0.88 (0.05)
			C	0.90 (0.08)	0.88 (0.05)
		Controls	A	0.77 (0.10)	0.96 (0.03)
			B	0.77 (0.10)	0.96 (0.03)
			C	0.77 (0.10)	0.96 (0.03)

A similar comparison of models 3 and 4 also addresses the issue of possibly differential accuracy by disease status, but now assuming that the laboratories have equal accuracy; the likelihood ratio test is χ^2 ^= 4.8 on 2 *df *(p = 0.09), suggesting that accuracy is not significantly related to disease status. This seems reasonable, because it is unlikely on biological grounds that errors in classifying this polymorphism would be related to disease [1].

Other comparisons between the models of Table 6 can address variation in accuracy across laboratories. For instance, a comparison of models 1 and 3 tests for equality between laboratories while assuming independence of accuracy and disease status, while a similar comparison of models 2 and 4 allows for a dependence of accuracy on disease. These tests give χ^2 ^= 8.6 on 4 *df *(p = 0.07) and χ^2 ^= 13.0 on 8 *df *(p = 0.11), assuming non-differential or differential test accuracy by disease status, respectively, thus giving weak evidence of inter-laboratory differences in accuracy. There is a suggestion that laboratory A has lower specificity, while laboratory C has lower sensitivity. However, these differences were not strongly supported by the likelihood ratio tests, which gave only borderline significance.

### Association of polymorphism with disease

Table 7 shows the results of models focussed on the association of the true genotype variable X with disease. The likelihood ratio test comparing models 5 and 6 (χ^2 ^= 8.0 on 4 *df*, p = 0.09) again weakly suggests that laboratory accuracy varies, and the pattern of parameter estimates is similar to those in Table 6. These models additionally estimate the conditional probabilities of X for given values of D (cases or controls), which in turn lead to their ORs. Model 5 gives estimates P(X = +|case) = 0.340 (SE = 0.066) and P(X = +|control) = 0.220 (SE = 0.048), where + and - indicate presence or absence of the Arg/Arg genotype respectively. This implies an OR of 1.83 (95%CI = 0.97, 3.46). The corresponding conditional probabilities in model 6 (with laboratories constrained to have equal accuracy) are 0.378 (SE = 0.072) and 0.237 (SE = 0.054), and an associated OR of 1.96. (95%CI = 1.02, 3.75). Given that there is no strong evidence of inter-laboratory differences in accuracy, the model 6 estimate of OR would be the preferred value.

**Table 7:** Results for latent class models focussing on association of latent exposure variable and disease

**Model**	**Log-likelihood**	**Lab**	**Sensitivity (SE)**	**Specificity (SE)**	**Estimated prevalence in cases (SE)**	**Estimated prevalence in controls (SE)**	**Odds ratio (CI)**
5	-439.87	A	0.94 (0.07)	0.90 (0.05)	0.34 (0.07)	0.22 (0.05)	1.83 (0.97–3.46)
		B	0.91 (0.11)	0.94 (0.06)			
		C	0.69 (0.10)	0.95 (0.03)			
6	-443.85	A	0.82 (0.07)	0.94 (0.03)	0.38 (0.07)	0.24 (0.05)	1.96 (1.02–3.75)
		B	0.82 (0.07)	0.94 (0.03)			
		C	0.82 (0.07)	0.94 (0.03)			

### Comparison with empirical results

Table 8 shows the empirical associations of laboratory results with disease. A comparison of models 7 and 8 assesses the possibility of different strength of association with disease by laboratory. Their likelihood ratio test (χ^2 ^= 4.78 on 4 *df*, p = 0.31) indicates no strong evidence for different associations by laboratory. The empirical estimates of OR are 2.48 (95%CI 1.10 – 5.60), 1.59 (95%CI 0.90 – 2.80), and 1.84 (95%CI 1.10 – 5.60), for laboratories A, B, C respectively.

**Table 8:** Results for empirical models focussing on association of laboratory values and disease

**Model**	**Log-likelihood**	**Lab**	**Observed prevalence in cases (SE)**	**Observed prevalence in controls (SE)**
7	-475.23	A	0.50 (0.08)	0.29 (0.05)
		B	0.34 (0.04)	0.24 (0.04)
		C	0.32 (0.08)	0.21 (0.05)
8	-477.62	A	0.37 (0.03)	0.24 (0.03)
		B	0.37 (0.03)	0.24 (0.03)
		C	0.37 (0.03)	0.24 (0.03)

## Discussion

Variation in measuring p53 expression has been recognized before, in the context of bladder cancer studies [26]. In this paper, we have illustrated the use of LCMs to evaluate the association of a genotype with cancer, while taking measurement error in the genotype into account. This approach is attractive for the rapidly increasing number of studies relating genetic traits to various diseases, but the models are also potentially applicable to a wide variety of other epidemiological investigations. The data discussed here came from several laboratories, but the same approach could be applied to studies where different methods are used to assess exposure or putative susceptibility to a risk factor, for instance questionnaires *vs*. medical records concerning risk determinants, self-report *vs*. proxy reports for dietary consumption, or different methods within the same laboratory.

We used several models to investigate the possibility of differential test accuracy by laboratory. These models can be fitted whenever the number of tests per subject is at least 2. For data with exactly 2 measurements, one can permit accuracy to be differential by disease status, but one cannot allow for differences between laboratories (or between methods in general). When there are 3 or more measurements per subject, one can examine the possibility of accuracy being differential by *both *disease and laboratory.

Use of LCMs when there is uncertainty about risk status is somewhat more feasible than when it is the disease status that may be misclassified. For the latter, one requires at least *three *measurements in order to estimate test accuracy and disease prevalence in a single population, or two measurements with data from two or more populations, assuming one can ignore the possibility of population by test interactions [7,27]. The particular case of two independent measurements in two populations was discussed in detail by Hui and Walter [28], this scenario being one of very few that admit a closed-form solution for the parameter estimates.

In analyses concerned with uncertainty about disease status, conditional independence of test errors is often assumed, but this assumption may not always be valid in practice. However, conditionally dependent errors can be included in the model if there are additional measurements available [29-32], but this presents an additional burden on the investigators, and it may not be feasible to include such additional measurements.

In contrast, when it is the risk factor that involves measurement error (as in the present example), the conditional independence assumption can be examined more easily, because of the more limited data requirements. In our data, we found no strong evidence of test accuracy being dependent on disease status, a reasonable finding given the underlying biology and the laboratory testing methods. We also tested the conditional independence assumption by adding terms such as AB|X to model 1. None of these terms was statistically significant, so there was no evidence of a departure from the conditional independence assumption. Drews et al. [33] describe an alternative latent class approach to situations with two measurements having non-differential and conditionally dependent errors, but the error correlations must either be known (somewhat unrealistic in practice) or at least taken to have given, fixed values.

We also found only weak evidence of differential accuracy by laboratory. However, with the given data (having only one result per woman for each laboratory), we were obliged to assume no subject-by-laboratory interaction, or in other words conditionally independent error rates by laboratories. This last interaction could be examined if there were replicated observations in the same laboratories.

The main objective of genetic studies of the type we have discussed is to obtain the best possible estimate of the OR between a polymorphism and disease. The LCMs we have used include all the available data, and yield maximum likelihood estimates of OR. While the test accuracy of laboratories is not a main focus, the latent class method does give estimates of accuracy as a useful by-product. Also, the evaluation of the fit of alternative LCMs that examine test accuracy provides guidance on the preferred way to allow for test inaccuracy when the polymorphism-disease association is addressed in later models. In our example, we found no convincing evidence of differential test accuracy by laboratory or disease status, which implied that the preferred model for the polymorphism OR should be the one (here, model 6) where accuracy is constrained to be equal in all laboratory-disease groups of data.

In our example, we exploited the existence of data from women whose samples had been analysed by more than one laboratory. Practicalities limited the number of samples where sufficient material was available for replicated testing, especially given the wide geographical spread of the participating laboratories. If there is primary interest in assessing test accuracy (as opposed to primary interest in the polymorphism OR), then an appropriate study should imply a sample design having more replicated observations with the analytic focus being on test variation between, and possibly within, laboratories.

## Conclusion

Our analysis provided an estimate of OR for the genotype-cancer association. Subject to the validity of the assumed model, this estimate enjoys the general properties of maximum likelihood estimates, including asymptotic unbiasedness and minimum variance. The model-based estimate also avoids the ambiguous and arbitrary choices that must be made between the various empirical estimates available when the genotype classifications disagree for some study subjects, as exemplified by the wide range of empirical ORs in Table 2, and as seen in the laboratory-specific estimates from model 7. Also, if the reliability of the data is low, the latent class OR estimate will tend to have a lower standard error and narrower confidence limits than the various empirical estimates. In our example, in which reliability was moderate or substantial, the latent class OR estimate was still somewhat more precise than the estimates for laboratories A and C. It was also statistically significant, whereas the empirical results for laboratories B and C were not.

An additional benefit of the LCM approach is that it yields estimates of the accuracy of the test method. In the absence of a definitive (i.e. an error-free gold standard) classification of exposure, the accuracy values can be used to calculate the predictive values associated with given test results, an attractive feature for clinical applications. The accuracy results may also help to identify deficiencies in data quality, e.g. from certain laboratories or observational methods.

The methods used here involved a binary risk factor, but they could easily be extended to cover multinomial exposures. [33,34] Extensions to the basic LCMs of Hui and Walter [28] have been proposed to allow for differential misclassification between cases and controls [33-35]; these approaches require specification of a covariate that defines two subgroups of cases and controls, across which the error rates of each observational method are assumed constant. Further extensions to allow for additional or continuous covariates can be envisaged. Potential difficulties with such extensions are the number of extra parameters required and the sparser distribution of the observations over a larger number of data cells when suitable covariates exist, or the unavailability of suitable covariates in other cases. The validity of the maximum likelihood parameter estimates and likelihood ratio tests to compare models might then be a concern. Others have commented [36,37] that likelihood methods may not perform well in distinguishing competing models in this context.

On the basis of the present re-assessment, we believe that previous attempts to compensate for the measurement error in the original study [1] may have led to over-estimates of the OR. A recent meta-analysis of all case-control studies on the association between p53 codon 72 polymorphism and cervical cancer risk indicated an average effect that was consistent with the LCM estimates presented here [38]. Likewise, the ORs we obtained in a recent case-control study specifically designed to verify the association, and which used improved methods to assess the polymorphism (involving less measurement error) [39], were consistent with the present latent class-based estimates.

## Authors' contributions

SDW developed the methodology presented in this paper, carried out the statistical analysis of the data, and drafted the manuscript. ELF has done research on the impact of exposure misclassification in cancer etiology studies and was responsible for the case-control study that generated the data used to illustrate the methods developed by SDW in the paper. Both authors read and approved the final manuscript.
